# Skill progression models in emergency medicine training: a narrative review and the development of the OASIS framework

**DOI:** 10.1186/s12245-025-00994-1

**Published:** 2025-10-01

**Authors:** Aaditya Katyal, Vimal Krishnan S

**Affiliations:** Department of Emergency Medicine, Kasturba Medical College, Manipal, Manipal Academy of Higher Education (MAHE), Manipal, Karnataka 576104 India

**Keywords:** Emergency medicine, Procedural training, Competencybased medical education, Skill acquisition, Supervision, Entrustment, OASIS framework, Simulationbased education, Peerassisted learning

## Abstract

**Background:**

Emergency medicine (EM) requires mastery of diverse procedural skills in unpredictable, high-acuity settings. While established educational models—Dreyfus, Miller’s pyramid, Peyton’s four-step approach, and Entrustable Professional Activities (EPAs)—offer useful concepts, none provide an integrated, EM-specific, stage-linked supervision roadmap.

**Objective:**

To review existing models of procedural skill acquisition and supervision relevant to EM and present the OASIS framework (Observe, Assist, Supervised performance, Independent performance, Supervise others), which integrates educational theory with explicit supervision criteria.

**Methods:**

A narrative review of MEDLINE, CINAHL, and Web of Science (1990–2025) was conducted using terms related to EM, procedural training, supervision, entrustment, and educational frameworks. Eligible sources included theoretical/conceptual works, empirical studies, systematic/narrative reviews, and consensus/guideline documents. Reference list checks and grey literature were also included.

**Results & discussion:**

Fifty-two publications met inclusion criteria: theoretical/conceptual works (*n* = 15), reviews (*n* = 12), empirical EM/acute care studies (*n* = 17), and consensus/guidelines (*n* = 8). Common gaps included: absence of explicit supervision stages, variability in entrustment decisions, limited faculty procedural competence, and inadequate bridging from simulation to clinical autonomy. Peer-assisted learning was underutilized. The OASIS framework addresses these gaps by mapping procedural skill progression to five explicit supervision levels, each with defined learning goals, preparatory simulation steps, clinical application, and assessment checkpoints. It integrates deliberate practice, mastery learning, non-technical skill development, and a structured peer-assisted learning component. Compared to existing models, OASIS operationalizes supervision behaviors, reduces variability in entrustment, and standardizes progression toward safe autonomy.

**Conclusion:**

OASIS offers an evidence-informed, practical roadmap for procedural supervision in EM, aligning technical and non-technical competencies with graded autonomy. Implementation requires faculty calibration and simulation access, with future multi-centre research needed to evaluate its educational and patient safety impact.

## Background

Emergency medicine (EM) is a rapidly evolving medical specialty that demands mastery of a broad spectrum of procedural skills performed in unpredictable, highstakes clinical environments. EM practitioners are required to competently execute lifesaving interventions such as endotracheal intubation, central venous catheter placement, chest tube thoracostomy, and pericardiocentesis, often under extreme time pressure and with limited resources. Compared to elective surgical or outpatient procedural settings, the emergency department (ED) poses unique challenges including patient instability, multitasking, and urgent decisionmaking where outcomes directly depend on timely and proficient intervention. As such, EM residency training emphasizes not only psychomotor proficiency but also clinical reasoning, situational awareness, leadership, and teamwork in managing complex, dynamic situations [1, 2].

The concept of graded responsibility formed the foundation of early clinical training paradigms, epitomized by Halsted’s “*see one*,* do one*,* teach one*” approach, which linked progression toward autonomy to demonstrated competence under supervision [3, 4]. While historically foundational, this model focused primarily on technical skill acquisition without incorporating cognitive or nontechnical competencies, nor did it specify explicit criteria for entrustment or graduated supervision. Over recent decades, competencybased medical education (CBME) frameworks have emerged, emphasizing outcomebased milestones, entrustment decisions, and progression dictated by demonstrated capability rather than time served [5, 6].

In EM, CBME principles are operationalized through the Accreditation Council for Graduate Medical Education (ACGME) Emergency Medicine Milestones, which provide structured behavioral anchors across patient care, medical knowledge, professionalism, and procedural domains [7, 8]. These milestones embed entrustment concepts, requiring faculty to judge when trainees can perform procedures with reduced or no supervision. However, their implementation is challenged by variability in faculty interpretation, inconsistent assessment practices, and the practical difficulty of sustained direct observation in the ED [9–11].

Procedural training in EM faces further barriers from decreased clinical exposure due to work hour restrictions, evolving models of care, the expansion of subspecialty services, and patient safety initiatives that restrict resident opportunities to perform rare but critical procedures such as pediatric airway management or emergency thoracotomy [12, 13]. Simulationbased education (SBE) has been adopted to address these limitations, providing safe, highfidelity environments for deliberate practice, exposure to uncommon scenarios, and rehearsal of crisis resource management skills [14–16]. While SBE improves early competence and confidence, several studies highlight the “competence–performance gap” observed when learners transition from simulation to independent clinical performance without structured, staged supervision [17, 18]. Compounding this, faculty skill decay in infrequently performed procedures raises concerns about supervision quality and underlines the importance of ongoing faculty development [19, 20].

The scope of EM procedural skills ranges from basic interventions (wound suturing, splint application) to advanced, highstakes techniques (ultrasoundguided vascular access, resuscitative thoracotomy). Competence entails not only executing the procedure but also understanding its indications, contraindications, necessary equipment, and possible complications [21]. Nontechnical skills—particularly leadership, communication, and teamwork—are critical determinants of procedural success and patient outcomes [22, 23].

Several educational models provide relevant conceptual underpinnings:

- Dreyfus model: Outlines progression from novice to expert through gradual transition from rulebound to intuitive, contextdriven performance [24, 25]. While conceptually robust, it does not prescribe how supervision should evolve across stages.
- Miller’s pyramid: Frames competence progression from “knows” to “does,” with a later “is trusted” apex highlighting entrustment [26, 27]. It specifies assessment layers but omits operational supervision guidance.
- Peyton’s four steps: Demonstration, deconstruction, comprehension, and performance; effective for early skill acquisition in simulation contexts [28, 29]. Its scalability to the ED environment is limited without adaptation.
- Entrustable Professional Activities (EPAs): Define clinical tasks that can be entrusted to trainees who have demonstrated readiness [30, 31]. While EMspecific EPAs frame essential procedures, interfaculty variability and limited stagelinked supervision detail constrain their reliability [32, 33].

Individually, these models enhance understanding of skill acquisition but fail to deliver a comprehensive, EMspecific supervision framework that integrates technical, cognitive, and nontechnical domains with clear entrustment decisions across developmental stages. To address this, we propose the OASIS framework—Observe, Assist, Supervised performance, Independent performance, Supervise others—which synthesizes elements from existing theories into an operational, stagelinked structure mapping learner progression to defined supervision levels, with embedded assessment checkpoints and integration of peerassisted learning.

## Methods

### Review design

This review used a narrative approach to explore procedural skill acquisition, supervision models, and progression frameworks in emergency medicine (EM) training. A narrative review was chosen instead of a systematic review to allow inclusion of theoretical frameworks, empirical research, and consensus or guideline documents. This method is especially suited for synthesizing diverse forms of evidence to inform the development of a new practical supervision framework.

### Data sources and search strategy

The primary literature search was conducted in MEDLINE (via PubMed), CINAHL (Cumulative Index to Nursing and Allied Health Literature), and Web of Science for publications from January 1990 to June 2025. This time frame encompasses the introduction of modern competencybased medical education (CBME) principles through to the latest innovations in EM training.

The search combined subject headings with freetext keywords across four themes:

1. Clinical context: “emergency medicine,” “emergency department,” “acute care”.
2. Skills and training: “procedural skills,” “procedural training,” “technical skills,” “simulation,” “competency-based medical education,” “CBME,” “clinical supervision”.
3. Educational models: “Dreyfus model,” “Miller’s pyramid,” “Peyton four-step,” “entrustable professional activities,” “EPAs”.
4. Assessment and progression: “entrustment decisions,” “milestones,” “peer-assisted learning”.

Boolean operators (AND, OR) and truncation (e.g., “competenc*”) were applied to broaden the search while maintaining relevance. An example strategy was:

(“emergency medicine” OR “acute care”) AND (“procedural skills” OR “procedural training”) AND (“competency-based medical education” OR “entrustable professional activities” OR “Dreyfus” OR “Miller’s pyramid” OR “Peyton”).

### Supplementary search methods

To supplement database searching, manual checks of reference lists from included works and key reviews were conducted to identify additional sources. Relevant grey literature, such as professional society guidelines, position statements, and consensus reports, was also screened where relevant to EM procedural supervision.

### Inclusion criteria

Publications were considered eligible if they met all three of the following criteria:

1. Described, evaluated, or made explicit reference to an educational model, framework, or supervision/entrustment strategy applicable to procedural skill training.
2. Related directly to EM or to comparable acute care contexts such as critical care or trauma surgery.
3. Published in English between 1990 and 2025.

### Exclusion criteria

Sources were excluded if they:

- Focused exclusively on procedural technique or device validation without an educational component.
- Addressed specialties without acute care relevance.
- Were opinion pieces unlinked to a formal model or empirical evidence.

### Screening and selection process

Search results were exported to Zotero v6 for deduplication. Two reviewers then independently screened titles and abstracts for relevance. Articles meeting inclusion criteria or where eligibility was unclear were retrieved in full text. Full-text eligibility was assessed collaboratively, with any disagreements resolved through discussion.

### Data extraction

Data were extracted for each study capturing:

- Publication year, country, and type (conceptual, empirical, review, consensus/guideline).
- Educational model/theory cited.
- Type and complexity of procedural skills discussed.
- Supervision or entrustment criteria described.
- Assessment tools or metrics referenced.
- Reported strengths, limitations, and context for EM training application.

### Synthesis approach

The included literature was grouped into themes:

1. Theoretical foundations of skill acquisition (Dreyfus, Miller, Peyton, EPAs).
2. Current procedural training practices and challenges in EM.
3. Supervision and entrustment frameworks.
4. Educational innovations (simulation, peer-assisted learning, faculty development) and identified gaps.

Insights from these themes were mapped to inform the structure of the proposed OASIS framework, linking observed training challenges to practical, stage-specific supervision strategies.

## Results & discussion

### Study selection

The structured databases search yielded 146 potential records, of which 112 remained after duplicate removal. A title and abstract screening phase excluded 64 articles that did not focus on procedural skill training, competency frameworks, or supervision models. Forty-eight studies met inclusion criteria upon full-text review, with four additional sources added through manual reference searches and grey literature exploration, resulting in 52 included publications. These consisted of 15 theoretical or conceptual papers, 12 narrative or systematic reviews, 17 empirical studies focused on emergency medicine or related acute care contexts, and 8 expert consensus or guideline documents. The publication dates ranged from 1990 to 2025, with a clear increase post-2010 corresponding to the broader adoption of competency-based medical education (CBME) and simulation-based training in emergency medicine.

**Fig. 1:**
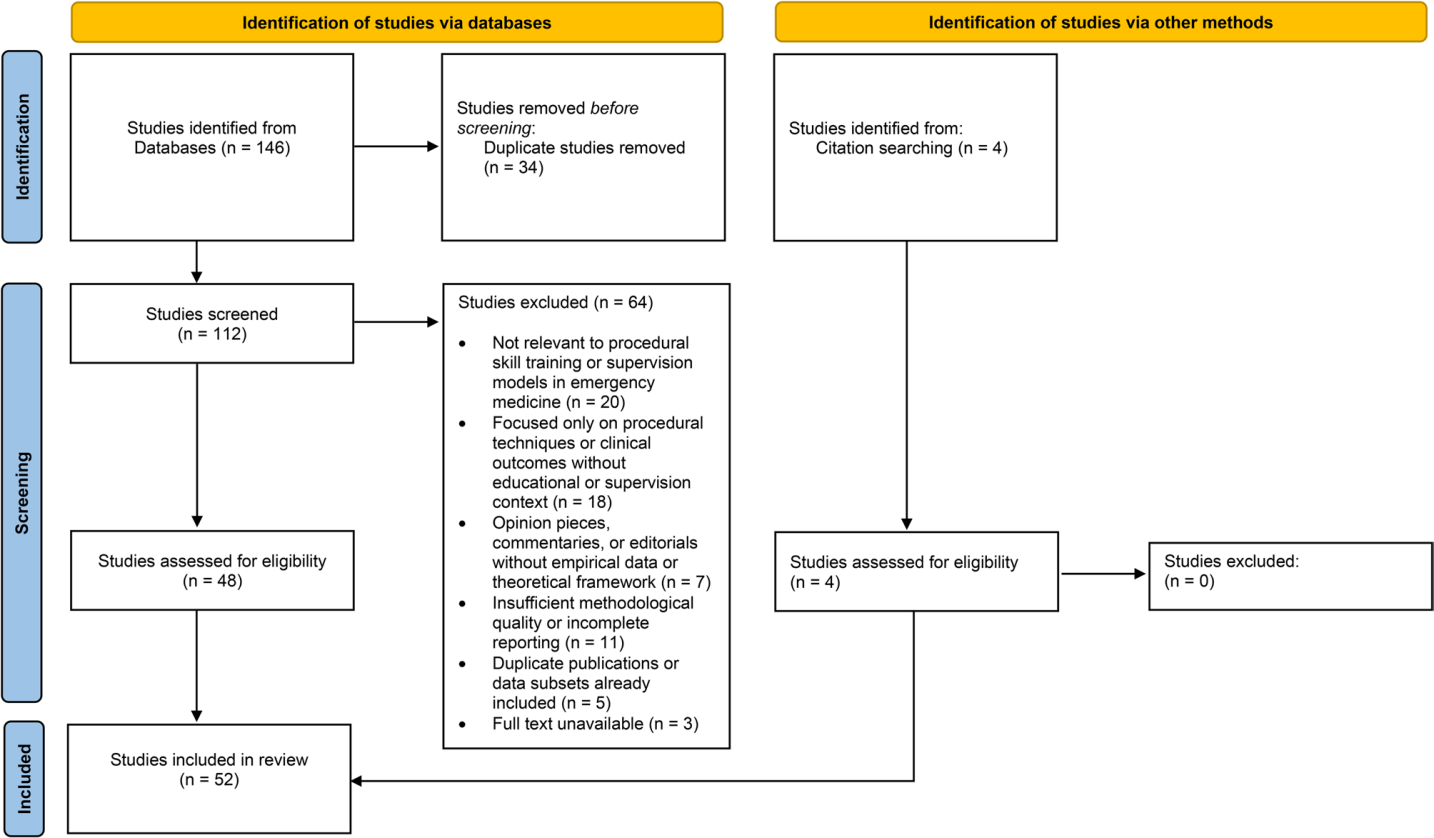
Study selection process

The study selection process is summarised in Fig. 1, outlining the number of records identified, screened, excluded, and retained for the final review.

### Theoretical models in emergency medicine procedural training

**Fig. 2:**
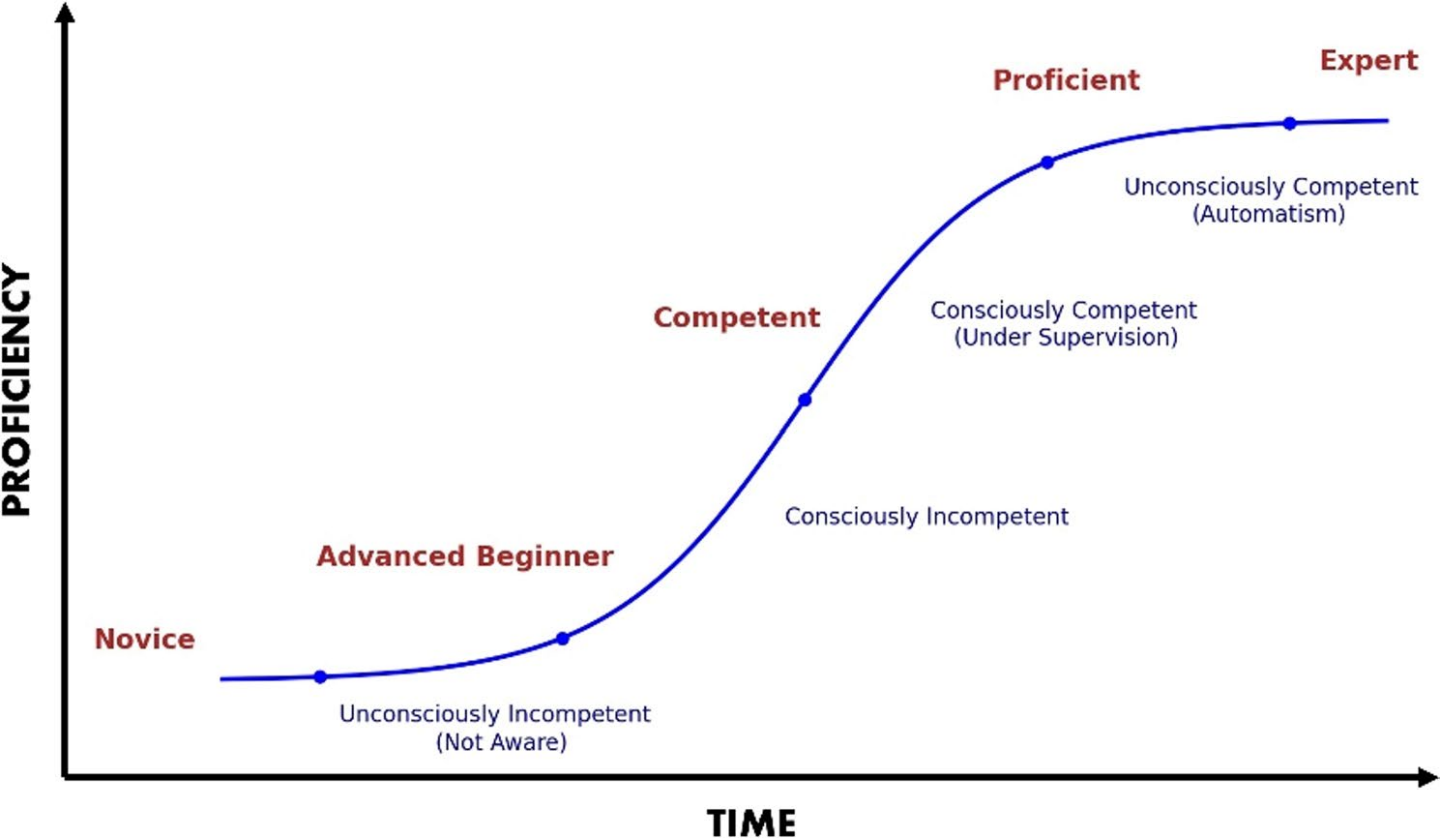
The Dreyfus model of skill acquisition

The five-stage Dreyfus model of skill acquisition was the most cited theoretical framework. It delineates learner progression from novice — relying on rules — through to expert, characterized by intuitive, context-based judgment (Fig. 2). Adaptations for clinical education have mapped these stages with observable learning behaviors [21, 24, 25]. While the model’s conceptual clarity has been appreciated in emergency medicine (EM), it lacks concrete guidance on how supervision intensity should adjust at different stages, limiting direct application for supervision of complex, urgent procedures [24].

Miller’s pyramid of clinical competence frames assessment progression from knowledge acquisition (“knows”), application (“knows how”), demonstration (“shows how”), to autonomous performance (“does”) [26]. Later extensions introduced the concept of “is trusted” to capture entrustment decisions [27]. In EM, Miller’s pyramid has underpinned milestone assessments and Entrustable Professional Activities (EPA) frameworks [7, 8, 30] but fails to operationalize how supervision should evolve with learner progression, leaving a gap in structured clinical oversight guidance.

Peyton’s four-step teaching approach — demonstration, deconstruction, comprehension, and performance — has robust evidence supporting its effectiveness for initial skill acquisition and retention, especially within simulation-based learning [28, 29]. However, its design assumes controlled learning environments with low learner-to-instructor ratios, which are at odds with the high cognitive load and multitasking found in emergency departments, limiting its direct scalability.

Entrustable Professional Activities provide a framework for translating competency milestones into discrete clinical tasks that can be entrusted to a trainee upon demonstration of adequate skill [30, 31]. Despite their promise, empirical EM studies reveal significant variability in faculty entrustment thresholds, particularly for low-frequency, high-stakes procedures, leading to inconsistent supervision and autonomy decisions [32, 33].

### Current procedural training challenges in emergency medicine

Several recent studies have highlighted a declining frequency of resident exposure to high-acuity, low-frequency (HALF) procedures in EM training programs [12, 13]. Safety regulations, increased subspecialization, and restricted duty hours have contributed to fewer hands-on opportunities for trainees in critical interventions such as surgical airway management and pericardiocentesis. This reduction potentially compromises the development of competence required for independent performance.

Simulation-based education has become the primary modality to mitigate these exposure gaps, offering deliberate practice and opportunities to rehearse crisis management in a risk-free environment [14–16]. Outcomes reported include improvements in procedure completion metrics, technique accuracy, and complication recognition. Nonetheless, research reveals a competence-to-performance gap when applying these acquired skills in real-world clinical settings, particularly when direct supervision and graduated autonomy are lacking [17, 18].

Faculty procedural proficiency itself varies, with a significant portion of EM educators reporting discomfort supervising rarely performed procedures [13]. This variation presents challenges in making consistent entrustment decisions and raises the need for ongoing faculty development focused on procedural skills and supervision methods.

While ACGME emergency medicine milestones have been widely adopted as a CBME tool, their application to procedural skills assessment remains inconsistent. Some programs continue to default to progression based on postgraduate year rather than competency demonstration, detracting from CBME’s core principles of outcome-driven education [5, 6, 9].

### Identified gaps in procedural supervision and training

From this synthesis of literature, four principal gaps emerge. First, there is an absence of an explicit, longitudinal supervision framework that links learner developmental stages to appropriate clinical oversight intensity in EM procedural training. Second, entrustment decisions vary significantly across faculty, influenced by subjective risk tolerance and situational pressures, leading to inconsistent trainee progression [32, 33]. Third, there is inadequate structuring for the critical transition between simulated practice and autonomous live procedural performance, contributing to skill decay and performance discrepancies [17, 18]. Fourth, despite clear benefits in other fields, peer-assisted learning is underutilized as a formal supervision and teaching strategy within EM procedural education [22].

### The OASIS framework: an explicit supervisory roadmap

**Fig. 3:**
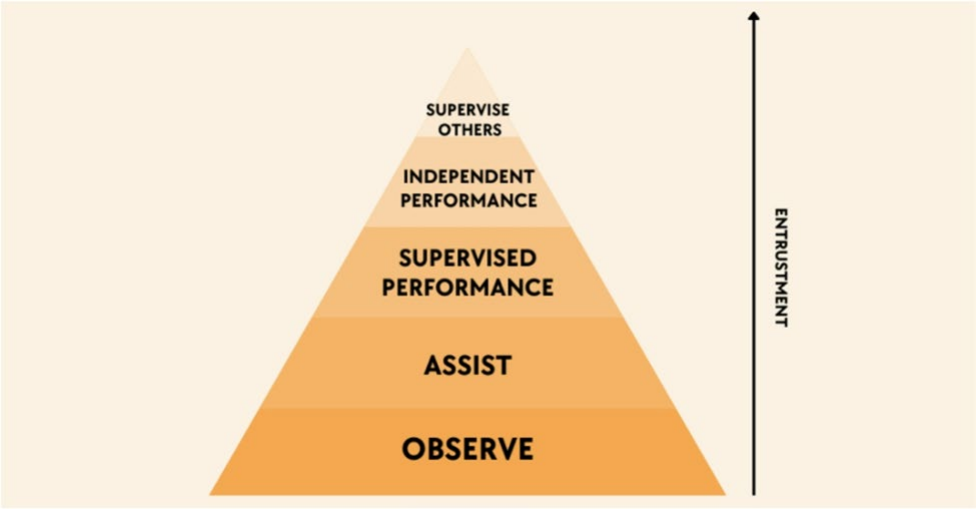
The OASIS Framework - An Explicit Supervisory Roadmap

The OASIS (Observe, Assist, Supervised performance, Independent performance, Supervise others) framework was developed to synthesize the strengths of foundational educational theories and address these documented gaps. It delineates five progressive supervision stages matched to learner competence development and workplace responsibility, with structured integration of simulation-based learning to bridge the competence-to-performance gap (Fig. 3*&* Table 1).

**Table 1:** OASIS stage progression framework rubric

Stage	Goal	Methods of Learning	Supervision Level	Assessment Criteria
Observe	Build foundational cognitive understanding of the procedure, including indications, contraindications, equipment, and potential complications.	Structured observation of expert procedures in clinical settings; annotated video review; faculty-led debriefs; discussion of procedural decision-making.	Full supervision — no hands-on activity; learner positioned as observer only.	Demonstrates accurate recall of procedural steps, indications, contraindications, and key safety considerations.
Assist	Gain initial hands-on familiarity with procedural components and develop psychomotor coordination.	Practice individual components on task trainers or part-task models; assist with procedural segments under direct faculty guidance in clinical settings.	Direct, “hands-on” supervision — supervisor maintains primary procedural control.	Confidently assists with assigned steps; explains procedural flow; demonstrates appropriate handling of instruments.
Supervised Performance	Achieve safe, consistent performance of the complete procedure under real-time oversight.	Perform full procedure in high-fidelity simulation; subsequently execute procedure on patients under direct faculty observation with immediate corrective feedback.	Direct supervision — supervisor scrubbed or present at bedside, ready to intervene instantly if required.	Completes procedure safely, efficiently, and to standard; integrates technical and situational decision-making; adapts appropriately to intra-procedural challenges.
Independent Performance	Execute the procedure autonomously with consistently safe and effective outcomes.	Perform procedure independently in patient care settings; engage in structured post-procedure debriefs and outcome reviews with faculty.	Distant or “gloves-off” supervision — supervisor immediately available but not physically present unless called.	Consistently completes procedure with high technical quality, sound clinical reasoning, and appropriate complication management.
Supervise Others	Consolidate mastery by teaching and supervising less experienced trainees, while ensuring patient safety.	Lead junior trainees through the procedure; provide real-time coaching, feedback, and correction under faculty oversight; reflect on teaching encounters.	Oversight shifted to trainee — responsible for supervision/teaching, with faculty monitoring trainee’s supervisory effectiveness.	Demonstrates ability to teach procedural steps clearly; corrects errors constructively; ensures safe trainee performance; receives positive feedback from learners and faculty.

The Observe stage aligns with the Dreyfus novice and Miller “knows” levels and incorporates Peyton’s initial demonstration and deconstruction steps. At this stage, learners gain foundational cognitive understanding through observing expert procedural performance in both clinical settings and high-fidelity simulation environments, participating in structured debriefing sessions aimed at consolidating knowledge of procedural indications, contraindications, equipment requirements, and potential complications.

The Assist stage corresponds to the Dreyfus advanced beginner and Miller “knows how” stages, paralleling Peyton’s comprehension phase. This stage incorporates a preparatory layer where trainees first practice relevant procedural sub-tasks and develop hand-eye coordination using task trainers and part-task simulation models. Subsequently, trainees engage directly with portions of the procedure under close clinical supervision, fostering psychomotor familiarity with procedural components along with guided feedback and real-time correction.

In the Supervised performance stage, learners perform entire procedures under direct faculty observation with immediate feedback incorporated. This phase aligns with the Dreyfus competent stage and Miller’s “shows how,” requiring procedural consistency, safety awareness, and real-time decision-making. A preparatory simulation layer precedes clinical performance, where learners complete the full procedure in high-fidelity simulated environments with faculty coaching to refine technique, decision-making, and error recovery before transitioning to patient care settings.

The Independent performance stage represents a transition to autonomous execution, reflecting the Dreyfus proficient and Miller “does” stages. Entrustment decisions grant learners permission to perform procedures unsupervised but with distant faculty availability, complete with post-procedure debriefing and reflection to support ongoing development. This stage emphasizes consistent competence demonstrated across both simulated and clinical contexts.

Finally, the Supervise others stage encompasses senior learners instructing and overseeing more junior colleagues in both simulation and clinical environments, supported by faculty mentorship. This stage fosters leadership and teaching skills while reinforcing procedural mastery, corresponding with the Dreyfus expert level and EPA entrustment apex. The inclusion of a structured peer-assisted learning component formalizes residents’ transition into educators, utilizing simulation laboratories for standardized instruction and clinical settings for supervised mentorship.

### Comparative analysis of OASIS versus established models

OASIS builds upon and extends the existing conceptual frameworks by providing detailed operational guidance for supervisory behaviors, thereby addressing critical limitations. While the Dreyfus model describes learner progression, it omits explicit supervision gradations; OASIS fills this gap by linking stages to clearly defined supervision levels and entrustment criteria. Miller’s pyramid conveys assessment levels but stops short of prescribing how clinical supervision should adapt; OASIS explicitly incorporates supervision intensity and stages of autonomy progression within the clinical environment.

Peyton’s technique is well validated for initial skill instruction but caters predominantly to simulated or controlled contexts and does not address continued supervised practice or gradual entrustment in clinical settings. OASIS preserves Peyton’s instructional strengths during early stages while incorporating the subsequent clinical supervision and independence phases.

EPAs bring a competency-based approach to entrustment by defining essential clinical tasks; however, their practical application suffers from inter-faculty variability and the absence of structured supervision stages to guide gradual learner progression. OASIS incorporates EPA principles but operationalizes them with explicit, stage-linked supervision and assessment guidelines, reducing subjective variability (Table 2).

**Table 2:** Crosswalk of OASIS supervision stages to existing competency models

OASIS Stage	Dreyfus Level	Miller Level	Peyton Step	EPA Link	Supervision Descriptor
Observe	Novice	Knows	Demonstration/Deconstruction	Preentrustment	Learner observes faculty performing the procedure with structured commentary; engages in postprocedure debrief to build foundational cognitive understanding before any handson activity.
Assist *(with preparatory task trainer step)*	Advanced Beginner	Knows How	Comprehension	Preentrustment	Preparatory layer: Learner first practises relevant procedural subtasks and handeye skills on task trainers to gain familiarity with instruments, ergonomics, and sequence. Clinical step: Learner then performs selected procedure components on patients under direct guidance, focusing on integrating psychomotor skills into realworld context.
Supervised Performance *(with preparatory simulated performance step)*	Competent	Shows How	Performance	Conditional entrustment	Preparatory layer: Learner completes the full procedure in a highfidelity simulated environment with faculty coaching to refine technique, decisionmaking, and error recovery. Clinical step: Learner subsequently performs the complete procedure on patients under direct faculty observation with immediate feedback and correction.
Independent Performance	Proficient	Does	—	Entrusted	Learner performs the procedure autonomously in clinical settings with distant supervision — faculty remains immediately available. Includes postprocedure debrief to reinforce learning.
Supervise Others	Expert	Does/Is Trusted	—	Full entrustment	Learner teaches and supervises junior colleagues performing the procedure under faculty oversight, reinforcing mastery and developing leadership and educator skills.

### Strengths of the OASIS framework

OASIS offers clarity and standardization in supervisor expectations and trainee progression, fostering consistent entrustment decisions and minimizing ambiguity. By integrating deliberate practice and mastery learning concepts, it supports progression only after verified competence, thus enhancing patient safety. The framework consciously integrates non-technical competencies including communication and teamwork, which are vital for successful procedural outcomes in emergency contexts.

Additionally, OASIS innovatively embeds peer-assisted learning into the advanced supervision stage, promoting sustainability of teaching resources and empowering senior residents as educators, which can improve overall departmental training capacity and learner confidence.

### Limitations and implementation challenges

Implementing OASIS in clinical programs requires substantial faculty development efforts to train supervisors in applying the framework reliably and in delivering constructive, formative feedback. Its early stages may depend heavily on access to simulation resources that are unavailable in some settings. Peer-assisted learning initiatives need formal oversight and preparation to maintain instructional consistency and quality.

Moreover, empirical validation remains necessary to confirm OASIS’s effectiveness across diverse training environments and to ensure its alignment and compatibility with existing CBME systems such as the ACGME Milestones.

### Future research directions

Future investigations should focus on multi-center, prospective evaluations of OASIS, measuring outcomes such as time to independent procedural performance, complication rates, patient safety metrics, and resident confidence. Studies should assess inter-rater reliability among faculty applying OASIS classification, as well as the framework’s impact on milestone attainment and curriculum development. Exploration of best practices for integrating peer-assisted learning and adaptation for resource-variable settings will further enhance OASIS’s applicability.

### Contextual implications for emergency medicine training

The OASIS framework directly responds to pressing competency challenges in EM procedural training, such as the documented reduction in resident procedural exposure and faculty supervision variability [12, 13]. By embedding explicit supervision strategies and assessment checkpoints, it promotes safe, measurable progression to independent practice. OASIS supports the EM educator’s role as a critical steward of both patient safety and trainee development, ensuring that residency training produces practitioners ready for the independent clinical environment.

## Conclusion

Procedural competence in emergency medicine demands far more than technical proficiency; it requires the integration of clinical reasoning, situational awareness, communication, teamwork, and an explicit progression towards independent practice. Existing models such as the Dreyfus framework, Miller’s pyramid, Peyton’s fourstep approach, and Entrustable Professional Activities provide valuable contributions to our understanding of skill acquisition and assessment, yet none individually delivers a practical, stagelinked supervision framework suited for the unique challenges of the emergency department.

The OASIS framework synthesizes the conceptual strengths of these models into a coherent, operational roadmap for procedural skill progression. By explicitly mapping supervision intensity to developmental stages, embedding deliberate practice and mastery learning principles, and incorporating nontechnical skills and peerassisted learning, OASIS offers a mechanism to standardize entrustment and safeguard both learner development and patient care.

Implementing OASIS will require faculty calibration, resource planning, and empirical validation across varied training environments. Its adoption holds the potential to improve procedural training consistency, accelerate safe autonomy, and enhance overall educational quality in emergency medicine. Future research should focus on evaluating its realworld impact on competency attainment, patient safety, and workforce readiness.

## Data Availability

No datasets were generated or analysed during the current study.

## References

[1] Hobgood C, Sherwood G, Frush K, et al. Teamwork training with nursing and medical students: does the method matter? Results of an interinstitutional, interdisciplinary collaboration. Qual Saf Health Care. 2010;19(6):e25. 20427311 10.1136/qshc.2008.031732

[2] Okuda Y, Bryson EO, DeMaria S Jr., et al. The utility of simulation in medical education: what is the evidence? Mt Sinai J Med. 2009;76(4):330–43. 19642147 10.1002/msj.20127

[3] Cameron JL. William Stewart Halsted. Ann Surg. 1997;225(5):445–8. 9193173 10.1097/00000658-199705000-00002 PMC1190776

[4] Halsted WS. The training of the surgeon. Bull Johns Hopkins Hosp. 1904;15:267–75.

[5] Frank JR, Snell LS, ten Cate O, et al. Competencybased medical education: theory to practice. Med Teach. 2010;32(8):638–45. 20662574 10.3109/0142159X.2010.501190

[6] Iobst WF, Sherbino J, ten Cate O, et al. Competencybased medical education in postgraduate medical education. Med Teach. 2010;32(8):651–6. 20662576 10.3109/0142159X.2010.500709

[7] Accreditation Council for Graduate Medical Education. Emergency medicine milestones. Chicago, IL: ACGME; 2015.

[8] Beeson MS, Carter WA, Christopher TA, et al. The development of the emergency medicine milestones. Acad Emerg Med. 2013;20(7):724–9. 23782404 10.1111/acem.12157

[9] Holmboe ES, Sherbino J, Long DM, Swing SR, Frank JR. The role of assessment in competency-based medical education. Med Teach. 2010;32(8):676–82. 20662580 10.3109/0142159X.2010.500704

[10] Agarwal A, Singh K, Bharti B, et al. Challenges in implementing competencybased medical education in india: A crosssectional study. Indian J Med Res. 2021;154(2):312–8.

[11] Van Melle E, Frank JR, Holmboe ES, et al. A core components framework for evaluating implementation of competencybased medical education programs. Acad Med. 2019;94(7):1002–9. 30973365 10.1097/ACM.0000000000002743

[12] Bandiera G, Leblanc C, Regehr G, et al. Education scholarship in emergency medicine part 1: innovating and improving teaching and learning. CJEM. 2014;16(Suppl 1):S1–5. 25027785 10.1017/s1481803500003146

[13] Clyne B, Doucet H, Brown L, et al. Maintaining procedural skills for academic emergency medicine faculty: A needs assessment. AEM Educ Train. 2021;5(1):e10648. 34853821 10.1002/aet2.10648 PMC8609535

[14] McGaghie WC, Issenberg SB, Cohen ER, Barsuk JH, Wayne DB. Does simulationbased medical education with deliberate practice yield better results than traditional clinical education? A metaanalytic comparative review of the evidence. Acad Med. 2011;86(6):706–11. 21512370 10.1097/ACM.0b013e318217e119 PMC3102783

[15] Cook DA, Brydges R, Zendejas B, Hamstra SJ, Hatala R. Mastery learning for health professionals using technologyenhanced simulation: a systematic review and metaanalysis. Acad Med. 2013;88(8):1178–86. 23807104 10.1097/ACM.0b013e31829a365d

[16] Kessler DO, Auerbach M, Pusic M, et al. Implementation and evaluation of simulationbased mastery learning for lumbar puncture. Simul Healthc. 2011;6(4):197–203. 21527870 10.1097/SIH.0b013e318216bfc1

[17] Rudolph JW, Simon R, Dufresne RL, Raemer DB. There’s no such thing as nonjudgmental debriefing: a theory and method for debriefing with good judgment. Simul Healthc. 2006;1(1):49–55. 19088574 10.1097/01266021-200600110-00006

[18] Beeson MS, Holmboe ES, Korte RC, et al. Initial validity analysis of the emergency medicine milestones. Acad Emerg Med. 2015;22(7):838–44. 26112031 10.1111/acem.12697

[19] Dreyfus HL, Dreyfus SE. Mind over machine: the power of human intuition and expertise in the era of the computer. New York, NY: Free; 1986.

[20] Dreyfus SE. The fivestage model of adult skill acquisition. Bull Sci Technol Soc. 2004;24(3):177–81.

[21] Carraccio CL, Benson BJ, Nixon LJ, Derstine PL. From the educational bench to the clinical bedside: translating the Dreyfus developmental model to the learning of clinical skills. Acad Med. 2008;83(8):761–7. 18667892 10.1097/ACM.0b013e31817eb632

[22] Vijayan ST, Kattuparambil JJ, Mani PT, et al. Juxtaposing pedagogical paradigms: the efficacy of peerassisted learning (PAL) versus facultyassisted learning (FAL) in the refinement of surgical proficiency. BMC Med Educ. 2025;25:290. 39984895 10.1186/s12909-025-06827-2 PMC11846272

[23] Miller GE. The assessment of clinical skills/competence/performance. Acad Med. 1990;65(9 Suppl):S63-7. 2400509 10.1097/00001888-199009000-00045

[24] Ten Cate O, Carraccio C, Damodaran A, et al. Entrustment decision making: extending miller’s pyramid. Acad Med. 2021;96(2):199–204. 33060399 10.1097/ACM.0000000000003800

[25] Peyton JWR. Teaching and learning in medical practice. Rickmansworth, Herts: Manticore Europe Ltd.; 1998.

[26] Giacomino K, Caliesch R, Sattelmayer KM. The effectiveness of Peyton’s fourstep teaching approach on skill acquisition: a systematic review and metaanalysis with integrated metaregression. PeerJ. 2020;8:e10129. 33083149 10.7717/peerj.10129 PMC7549471

[27] Ten Cate O. Entrustable professional activities: A process to translate competencies into clinical practice. Acad Med. 2005;80(4 Suppl):S1S9.

[28] Association of American Medical Colleges. Core entrustable professional activities for entering residency. Washington, DC: AAMC; 2014.

[29] Chahine S, Regehr G, Brydges R. Challenges in implementing EPAs in clinical training: a qualitative inquiry. Med Educ. 2021;55(4):503–13.

[30] Ericsson KA. Deliberate practice and acquisition of expert performance: a general overview. Acad Emerg Med. 2008;15(11):988–94. 18778378 10.1111/j.1553-2712.2008.00227.x

[31] Caverzagie KJ, Cooney TG, Hemmer PA, et al. The development of entrustable professional activities for internal medicine residency training: a report from the education redesign committee of the alliance for academic internal medicine. Acad Med. 2015;90(4):479–84. 25406600 10.1097/ACM.0000000000000564

[32] Warm EJ, Mathis BR, Held JD, et al. Entrustment and mapping of EPAs to milestones for internal medicine residents. J Grad Med Educ. 2014;6(4):763–9.

[33] Ten Cate O, Hart D, Ankel F, et al. Entrustment decision making in clinical training. Acad Med. 2016;91(2):191–8. 26630606 10.1097/ACM.0000000000001044

